# Impact of pemafibrate in patients with metabolic dysfunction‐associated steatotic liver disease complicated by dyslipidemia: A single‐arm prospective study

**DOI:** 10.1002/jgh3.13057

**Published:** 2024-04-02

**Authors:** Hiroki Ono, Masanori Atsukawa, Akihito Tsubota, Taeang Arai, Kenta Suzuki, Tetsuyuki Higashi, Michika Kitamura, Kaori Shioda‐Koyano, Tadamichi Kawano, Yuji Yoshida, Tomomi Okubo, Korenobu Hayama, Norio Itokawa, Chisa Kondo, Mototsugu Nagao, Masato Iwabu, Katsuhiko Iwakiri

**Affiliations:** ^1^ Division of Gastroenterology and Hepatology Nippon Medical School Tokyo Japan; ^2^ Project Research Units (PRU) Research Center for Medical Science The Jikei University School of Medicine Tokyo Japan; ^3^ Division of Gastroenterology Nippon Medical School Chiba Hokusoh Hospital Chiba Japan; ^4^ Division of Endocrinology, Diabetes and Metabolism Nippon Medical School Tokyo Japan

**Keywords:** homeostasis model assessment‐insulin resistance, liver fibrosis, metabolic dysfunction‐associated steatotic liver disease, non‐alcoholic fatty liver disease, pemafibrate

## Abstract

**Background and Aim:**

This study aimed to clarify the efficacy and safety of 48‐week pemafibrate treatment in patients with metabolic dysfunction‐associated steatotic liver disease (MASLD) complicated by dyslipidemia.

**Methods:**

A total of 110 patients diagnosed with MASLD complicated by dyslipidemia received pemafibrate at a dose of 0.1 mg twice daily for 48 weeks.

**Results:**

The participants were 54 males and 37 females, with a median age of 63 (52–71) years. Besides improvement in lipid profile, significant reductions from baseline to 48 weeks of treatment were found in liver‐related enzymes, such as aspartate aminotransferase, alanine aminotransferase (ALT), gamma‐glutamyl transpeptidase, and alkaline phosphatase (*P* < 0.001 for all). A significant decrease in the homeostasis model assessment‐insulin resistance (HOMA‐IR) was observed in patients with insulin resistance (HOMA‐IR ≥ 2.5) (4.34 at baseline to 3.89 at Week 48, *P* < 0.05). Moreover, changes in ALT were weakly correlated with those in HOMA‐IR (*r* = 0.34; *p* < 0.05). Regarding noninvasive liver fibrosis tests, platelets, Wisteria floribunda agglutinin‐positive Mac‐2‐binding protein, type IV collagen 7s, and the non‐alcoholic fatty liver disease fibrosis score significantly decreased from baseline to Week 48. Most adverse events were Grades 1–2, and no drug‐related Grade 3 or higher adverse events were observed.

**Conclusion:**

This study demonstrated that 48‐week pemafibrate administration improved liver‐related enzymes and surrogate marker of liver fibrosis in patients with MASLD. The improvement of insulin resistance by pemafibrate may contribute to the favorable effect on MASLD complicated by dyslipidemia.

## Graphical abstract

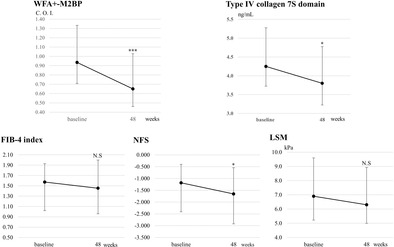

## Introduction

Metabolic dysfunction‐associated steatotic liver disease (MASLD), formerly called non‐alcoholic fatty liver disease (NAFLD), is the most common chronic liver disease, with a morbidity rate of ≥25%.^1^, ^2^, ^3^ Some patients with MASLD have liver necrosis and fibrosis, which develop into liver cirrhosis and hepatocellular carcinoma.^4^ MASLD is a multifactorial disease intricately linked to genetic, environmental, and metabolic factors.^5^ Specifically, metabolic factors are closely associated with MASLD onset/progression. Indeed, the prevalence of concomitant dyslipidemia is high (42–72%), and hypertriglyceridemia promotes liver fibrosis progression in patients with MASLD.^6^, ^7^, ^8^ Currently, a medical agent specific to MASLD is nonexistent; therefore, primary treatment includes improvements in lifestyle‐related aspects, such as dietary and exercise therapies.^9^, ^10^, ^11^ Meanwhile, when patients are afflicted with metabolic comorbidities, such as diabetes mellitus, hypertension, and dyslipidemia, pharmacotherapy is recommended to control these comorbidities.^12^, ^13^, ^14^

Glucagon‐like peptide 1 receptor agonists (GLP‐1 RAs)^15^, ^16^, ^17^ and sodium‐glucose cotransporter 2 inhibitors (SGLT2‐Is),^18^, ^19^, ^20^, ^21^ both of which are antidiabetic agents, have been reported to improve liver function test values and hepatic histological findings in patients with MASLD. Furthermore, pemafibrate, a selective peroxisome proliferator‐activated receptor α modulator available for hypertriglyceridemia treatment in Japan, has been reported to have ameliorating effects on biochemistry and hepatic histology in patients with MASLD complicated by dyslipidemia.^22^, ^23^, ^24^, ^25^, ^26^ Peroxisome proliferator‐activated receptors (PPARs) are a transcription factor that belongs to nuclear receptor subfamily 1 group C (NR1C) and are classified into three subtypes: PPARα, γ, and σ (NR1C1, NR1C3, and NR1C2, respectively). PPARs form heterodimers with retinoid X receptors and bind to PPAR‐responsive regulatory elements for transcriptional control. PPARα is primarily found in the liver and decreases triglyceride levels through the following primary mechanisms: increased β‐oxidation in the liver and increased lipoprotein lipase activity in the blood.^27^, ^28^ In Japan, Phase 2 and 3 clinical trials revealed that the triglyceride‐lowering action of pemafibrate was more potent than that of fenofibrate (a PPARα agonist) and that the former was safer for liver and kidney than the latter. Additionally, pemafibrate improved serum liver enzyme levels (ALT and γ‐GTP), whereas fenofibrate did not.^29^, ^30^

Several retrospective studies have reported that pemafibrate improved liver inflammation and fibrosis in patients with MASLD complicated by hypertriglyceridemia or dyslipidemia.^24^, ^25^, ^26^ These improvements may be attributed to the effect of pemafibrate on hepatic lipid metabolism and its direct ameliorative effect on liver inflammation/fibrosis; however, these mechanisms have not yet been fully understood. Moreover, a subgroup analysis of a Phase 3 clinical trial reported insulin resistance improvement due to pemafibrate.^31^ However, there have been no reports evaluating the relationship between liver injury and insulin resistance improvements in patients with MASLD treated with pemafibrate. Therefore, this prospective study aimed to evaluate the efficacy of 48‐week pemafibrate administration on liver injury, lipid metabolism, and insulin resistance, including its safety, and clarify the association between liver injury improvement and lipid metabolism or insulin resistance improvement in patients with MASLD complicated by dyslipidemia.

## Methods

### Study design

This was a prospective study to evaluate the effectiveness and safety of 48‐week pemafibrate treatment (UMIN registration no. 000049937), which registered 110 consecutive patients with MASLD newly received pemafibrate for dyslipidemia including abnormally high levels of triglyceride (≥150 mg/dL) and/or low‐density lipoprotein (LDL) cholesterol (≥140 mg/dL) at Nippon Medical School Hospital and Nippon Medical School Chiba Hokusoh Hospital between June 2021 and October 2022. Pemafibrate was orally administered at a dose of 0.1 mg twice daily. All patients fulfilled the diagnosis criteria for MASLD^32^ and did not meet the exclusion criteria. MASLD was diagnosed based on the following conditions: (i) the presence of hepatic steatosis on liver biopsy specimens or intrahepatic fat deposition on ultrasonography; (ii) the presence of at least one of cardiometabolic risk factors (i.e. the components of metabolic syndrome); (iii) weekly alcohol intake of <210 g for male and <140 g for female (average daily alcohol intake of <30 g for male and <20 g for female); and (iv) absence of any other specific causes of steatosis, as confirmed by medical histories, laboratory data, and imaging examinations. The main exclusion criteria were as follows: (i) age <20 years; (ii) initiation of other antilipidemic or diabetic agents and effective treatment for MASLD (such as vitamin E, pioglitazone, SGLT2‐Is, and GLP‐1 RAs) within 12 weeks prior to and during pemafibrate treatment; (iii) decompensated cirrhosis; (iv) biliary obstruction; (v) cholelithiasis; (vi) pregnancy or lactation; and (vii) ciclosporin or rifampicin administration. Antilipidemic and diabetic agents that had been administered for more than 12 weeks before pemafibrate treatment were not changed during the study period. There were no changes in exercise/dietary habits as well as in the aforementioned agents after starting pemafibrate during the study period. This study was performed in accordance with the ethical guidelines of the 2013 Declaration of Helsinki and was approved by the Ethics Committee of Nippon Medical School Hospital (approval number: B‐2020‐319). All patients provided written informed consent prior to study inclusion.

### Clinical and laboratory data

History, physical, and laboratory examinations were conducted at baseline and every 12 weeks during the 48‐week study period. Laboratory evaluation included complete blood count, routine liver‐related biochemistry (aspartate aminotransferase [AST], alanine aminotransferase [ALT], gamma‐glutamyl transpeptidase [γ‐GTP], alkaline phosphatase [ALP], and albumin), fasting lipids (total cholesterol, triglycerides, HDL cholesterol, and LDL cholesterol), and diabetes‐related tests (fasting plasma glucose, HbA1c, and immunoreactive insulin [IRI]). Homeostasis model assessment‐insulin resistance (HOMA‐IR) was calculated using the following equation: HOMA‐IR = fasting IRI (μU/mL) × plasma glucose (mg/dL)/405.^33^ HOMA‐IR was evaluated in 45 patients who were not receiving oral or injectable hypoglycemic medications, because HOMA‐IR could not be accurately assessed during the administration of these drugs. Insulin resistance was defined as HOMA‐IR ≥ 2.5. BMI was calculated as weight (kg) divided by the square of height (m^2^). Type IV collagen 7s domain and Wisteria floribunda agglutinin‐positive Mac‐2‐binding protein (WFA^+^‐M2BP) were measured as liver fibrosis markers. The Fibrosis‐4 (FIB‐4) index and the NAFLD fibrosis score (NFS) were calculated to evaluate the degree of liver fibrosis, as reported previously.^34^, ^35^ Liver stiffness measurement (LSM) and controlled attenuation parameter (CAP) were measured through transient elastography using FibroScan 502 equipped with the M‐probe (Echosens SA, Paris, France). Patients were divided into the advanced and less‐advanced fibrosis groups with a cutoff value of 192 × 10^3^/μL for platelets^36^ and high‐ and low‐albumin groups with a cutoff value of 4.0 g/dL.^37^ Responders to pemafibrate were defined as patients with a decrease in ALT level of 30% or more from baseline to Week 48.

### Statistical analyses

Continuous variables were presented as medians and interquartile ranges in parentheses. The Wilcoxon signed‐rank test was used to compare two paired groups. The Friedman test followed by post hoc pairwise comparisons was used to evaluate time‐course changes in continuous variable levels among multiple time points. Continuous variables with skewed distributions were compared between two groups using the Mann–Whitney *U*‐test, while categorical variables were compared using the Fisher's exact test. Spearman's rank correlation coefficient was used to analyze the correlations between continuous variables. All statistical analyses were performed using the Excel Statistics 2015 software (SSRI, Tokyo). The level of statistical significance was set at *P* < 0.05.

## Results

### Patient characteristics

Of the 110 patients, 91 completed 48‐week pemafibrate treatment as scheduled and were evaluated for the treatment effectiveness: specifically, time‐course changes in drug‐related parameters throughout the 48‐week study period. Table 1 summarizes the baseline patient characteristics. There were 54 males and 37 females, with a median age of 63 (52–71) years. The median BMI was 27.1 (24.1–30.1) kg/m^2^. Seventy‐four patients were diagnosed with hepatic steatosis by ultrasonography and 17 by liver biopsy. Forty‐three (47.3%) patients had type 2 diabetes. Concomitant medications with potential effects on MASLD were as follows: SGLT2‐Is (*n* = 24, 26.4%), GLP‐1 RAs (*n* = 6, 6.6%), and vitamin E (*n* = 3, 3.3%). The remaining 19 patients discontinued the treatment prematurely due to several reasons: adverse events (*n* = 3), long home‐to‐hospital distance (*n* = 8), unknown (*n* = 7), and transfer for surgery (*n* = 1). Treatment safety was evaluated for all 110 patients.

**Table 1:** Baseline characteristics of the 91 patients

Factors	*n* = 91
Age (year)	63 (52–71)
Gender (male/female)	54/37
Body weight (kg)	71.1 (62.0–81.3)
BMI (kg/m^2^)	27.1 (24.1–30.1)
Diagnostic methods for hepatic steatosis (ultrasonography/liver biopsy)	74/17
Platelets (×10^3^/mm^3^)	217 (176–257)
AST (U/L)	36 (28–49)
ALT (U/L)	52 (34–76)
γ‐GTP (U/L)	56 (40–123)
ALP (U/L)	118 (71–232)
Serum albumin (g/dL)	4.4 (4.2–4.7)
Triglyceride (mg/dL)	198 (159–274)
Total cholesterol (mg/dL)	223 (193–252)
LDL cholesterol (mg/dL)	137 (112–168)
HDL cholesterol (mg/dL)	47 (40–56)
Fasting plasma glucose (mg/dL)	119 (100–139)
IRI (μU/mL)	13.3 (9.9–16.7)
HOMA‐IR	3.75 (2.59–5.10)
Type 2 diabetes mellitus (presence/absence)	43/48
Type IV collagen 7S domain (ng/mL)	4.3 (3.7–5.3)
WFA^+^‐M2BP (C.O.I.)	0.94 (0.71–1.33)
FIB‐4 index	1.57 (1.02–1.93)
NFS	−1.183 (−2.413 to −0.400)
LSM (kPa)	6.9 (5.2–9.6)
CAP (dB/m)	306 (272–344)
Concomitant medications	
DPP4 antagonist	20 (22.0)
SGLT2 inhibitor	24 (26.4)
Metformin	12 (13.2)
GLP‐1 receptor agonist	6 (6.6)
Statin	7 (7.7)
Ezetimibe	1 (1.1)
Vitamin E	3 (3.3)
Calcium channel blocker	21 (23.1)
Angiotensin II receptor blocker	25 (27.5)
Beta‐blocker	2 (9.5)

Data are presented as numbers or medians (interquartile ranges). Numbers in parentheses refer to the percentage of patients.

ALP, alkaline phosphatase; ALT, alanine aminotransferase; AST, aspartate aminotransferase; BMI, body mass index; CAP, controlled attenuation parameter; DPP4, dipeptidyl peptidase‐4; FIB‐4 index, Fibrosis‐4 index; GLP‐1, Glucagon‐like peptide‐1; HDL, high‐density lipoprotein; HOMA‐IR, homeostasis model assessment‐insulin resistance; IRI, immunoreactive insulin; LDL, low‐density lipoprotein; LSM, liver stiffness measurement; NFS, NAFLD (non‐alcoholic fatty liver disease) fibrosis score; SGLT2, sodium‐glucose cotransporter 2; WFA^+^‐M2BP, Wisteria floribunda agglutinin‐positive Mac‐2‐binding protein; γ‐GTP, gamma‐glutamyl transpeptidase.

### Time‐course changes in laboratory, body weight, and CAP

**Figure 1:**
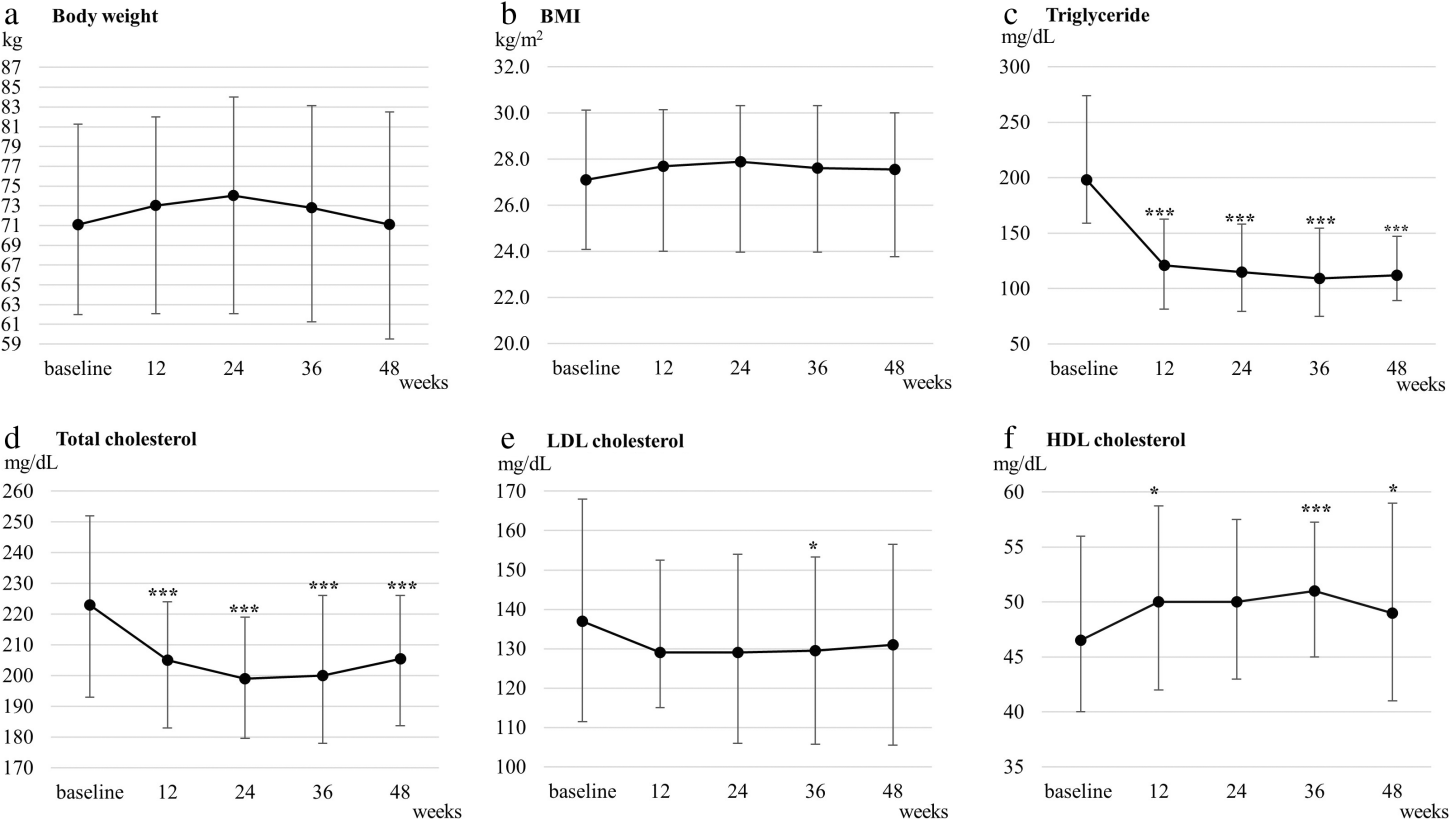
Changes from baseline in (a) body weight, (b) body mass index (BMI), (c) triglyceride, (d) total cholesterol, (e) low‐density lipoprotein (LDL) cholesterol, and (f) high‐density lipoprotein (HDL) cholesterol in patients treated with pemafibrate for 48 weeks. Error bars denote interquartile ranges. *P < 0.05 *versus* baseline. **P < 0.01 *versus* baseline. ***P < 0.001 *versus* baseline.

Regarding fasting lipids, significant decreases in triglyceride and total cholesterol (*P* < 0.001 for both), and a significant increase in HDL cholesterol (*P* < 0.05) were noted at Week 12, compared with baseline levels (Table 2 and Fig. 1). These significant changes were maintained until Week 48. In contrast, no significant decreases in LDL cholesterol were observed during the study period (except Week 36).

**Table 2:** Changes in clinical characteristics in the 91 patients who received pemafibrate for 48 weeks.

Pemafibrate therapy							
	Baseline	12 weeks	* *P* value	24 weeks	* *P* value	48 weeks	* *P* value
Body weight (kg)	71.1	73.0	0.90	74.0	0.69	71.1	0.34
	(62.0–81.3)	(62.1–82.0)		(62.1–84.0)		(59.5–82.5)	
BMI (kg/m^2^)	27.1	27.7	0.90	27.9	0.76	27.5	0.23
	(24.1–30.1)	(24.0–30.1)		(24.0–30.3)		(23.8–30.0)	
AST (U/L)	36	32	0.12	31	0.16	33	<0.001
	(28–49)	(24–46)		(25–45)		(22–42)	
ALT (U/L)	52	37	<0.001	35	<0.001	34	<0.001
	(34–76)	(23–59)		(22–53)		(20–54)	
γ‐GTP (U/L)	56	33	<0.001	38	<0.001	32	<0.001
	(40–123)	(21–61)		(21–57)		(22–53)	
ALP (U/L)	118	58	<0.001	57	<0.001	59	<0.001
	(71–232)	(43–103)		(43–74)		(45–72)	
Triglyceride (mg/dL)	198	121	<0.001	115	<0.001	112	<0.001
	(159–274)	(81–163)		(80–158)		(89–147)	
Total cholesterol (mg/dL)	223	205	<0.001	199	<0.001	206	<0.001
	(193–252)	(183–224)		(180–219)		(184–226)	
LDL cholesterol (mg/dL)	137	129	0.88	129	0.19	131	0.55
	(112–168)	(115–153)		(106–154)		(106–157)	
HDL cholesterol (mg/dL)	47	50	<0.05	50	0.43	49	<0.05
	(40–56)	(42–59)		(43–58)		(41–59)	
Fasting plasma glucose (mg/dL)	119	115	0.71	116	0.63	113	0.97
	(100–139)	(102–134)		(101–133)		(102–137)	
IRI (μU/mL)	13.3	—	—	—	—	11.3	0.05
	(9.9–16.7)					(8.9–15.7)	
HOMA‐IR	3.75	—	—	—	—	3.10	0.07
	(2.59–5.10)					(2.19–5.12)	
Patients with HOMA‐IR ≥2.5	4.34	—	—	—	—	3.89	<0.05
	(3.29–5.49)					(2.62–5.27)	
Patients with HOMA‐IR < 2.5	2.00	—	—	—	—	2.04	0.17
	(1.12–2.13)					(1.14–2.85)	
Platelets (×10^3^/μL)	217	—	—	—	—	237	<0.001
	(176–257)					(206–283)	
Serum albumin (g/dL)	4.4	—	—	—	—	4.5	<0.05
	(4.2–4.7)					(4.3–4.7)	
WFA^+^‐M2BP (C.O.I.)	0.94	—	—	—	—	0.65	<0.001
	(0.71–1.33)					(0.46–1.03)	
Type IV collagen 7S domain (ng/mL)	4.3	—	—	—	—	3.8	<0.05
	(3.7–5.3)					(3.2–4.8)	
FIB‐4 index	1.57	—	—	—	—	1.45	0.07
	(1.02–1.93)					(0.96–2.00)	
NFS	−1.183	—	—	—	—	−1.655	<0.01
	(−2.413 to −0.400)					(−2.922 to −0.543)	
LSM (kPa)	6.9	—	—	—	—	6.3	0.13
	(5.2–9.6)					(5.0–8.9)	
CAP (dB/m)	306	—	—	—	—	307	0.42
	(272–344)					(284–346)	

* *Versus* baseline.

Data are presented as medians (interquartile ranges).

ALP, alkaline phosphatase; ALT, alanine aminotransferase; AST, aspartate aminotransferase; BMI, body mass index; CAP, controlled attenuation parameter; FIB‐4 index, Fibrosis‐4 index; HDL, high‐density lipoprotein; HOMA‐IR, homeostasis model assessment‐insulin resistance; IRI, immunoreactive insulin; LDL, low‐density lipoprotein; LSM, liver stiffness measurement; NFS, NAFLD (non‐alcoholic fatty liver disease) fibrosis score; WFA^+^‐M2BP, Wisteria floribunda agglutinin‐positive Mac‐2‐binding protein; γ‐GTP, gamma‐glutamyl transpeptidase.

**Figure 2:**
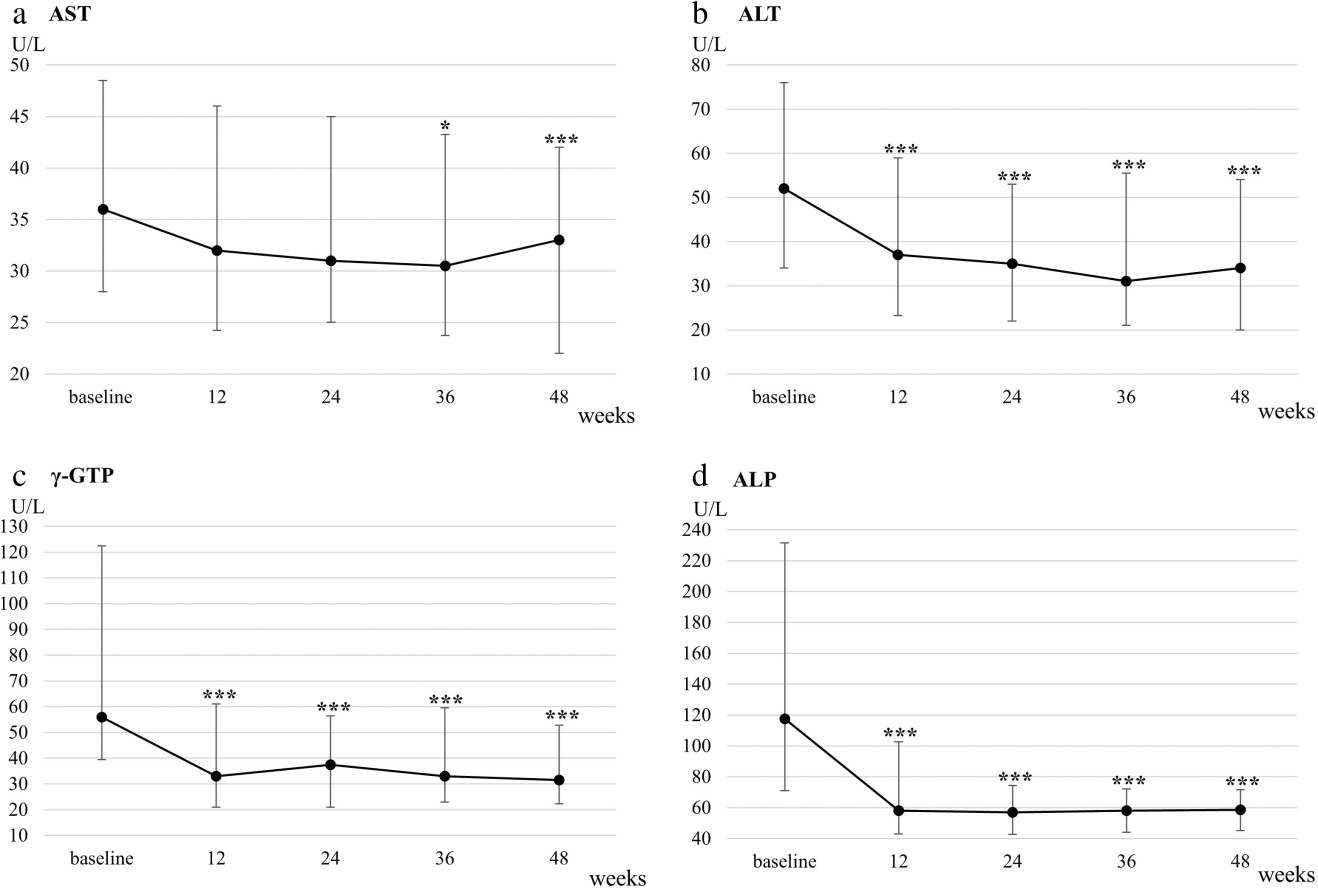
Changes in (a) aspartate aminotransferase (AST), (b) alanine aminotransferase (ALT), (c) gamma‐glutamyl transpeptidase (γ‐GTP), and (d) alkaline phosphatase (ALP) in patients treated with pemafibrate for 48 weeks. Error bars denote interquartile ranges. *P < 0.05 *versus* baseline. **P < 0.01 *versus* baseline. ***P < 0.001 *versus* baseline.

Concerning liver‐related biochemistry, ALT, γ‐GTP, and ALP levels significantly decreased from baseline throughout the 48‐week treatment period (*P* < 0.001 for all; Table 2 and Fig. 2). Comparing baseline characteristics of responders and non‐responders, AST (*P* < 0.01), ALT (*P* < 0.01), and γ‐GTP (*P* < 0.01) were significantly higher in responders than non‐responders (Table S1, Supporting information). IRI and HOMA‐IR decreased from baseline to Week 48, although these decreases were marginally significant (*P* = 0.05 and 0.07, respectively). When limited to patients with insulin resistance (HOMA‐IR ≥ 2.5), HOMA‐IR significantly improved at Week 48 (*P* < 0.05; Table 2). There were no significant changes in body weight and BMI from baseline throughout the 48 weeks (Fig. 1). Changes in BMI from baseline to Week 48 were not correlated with those in ALT (*r* = 0.02; *P* = 0.84), γ‐GTP (*r* = 0.02; *P* = 0.84), and HOMA‐IR (*r* = 0.25; *P* = 0.12) (Fig. S1).

**Figure 3:**
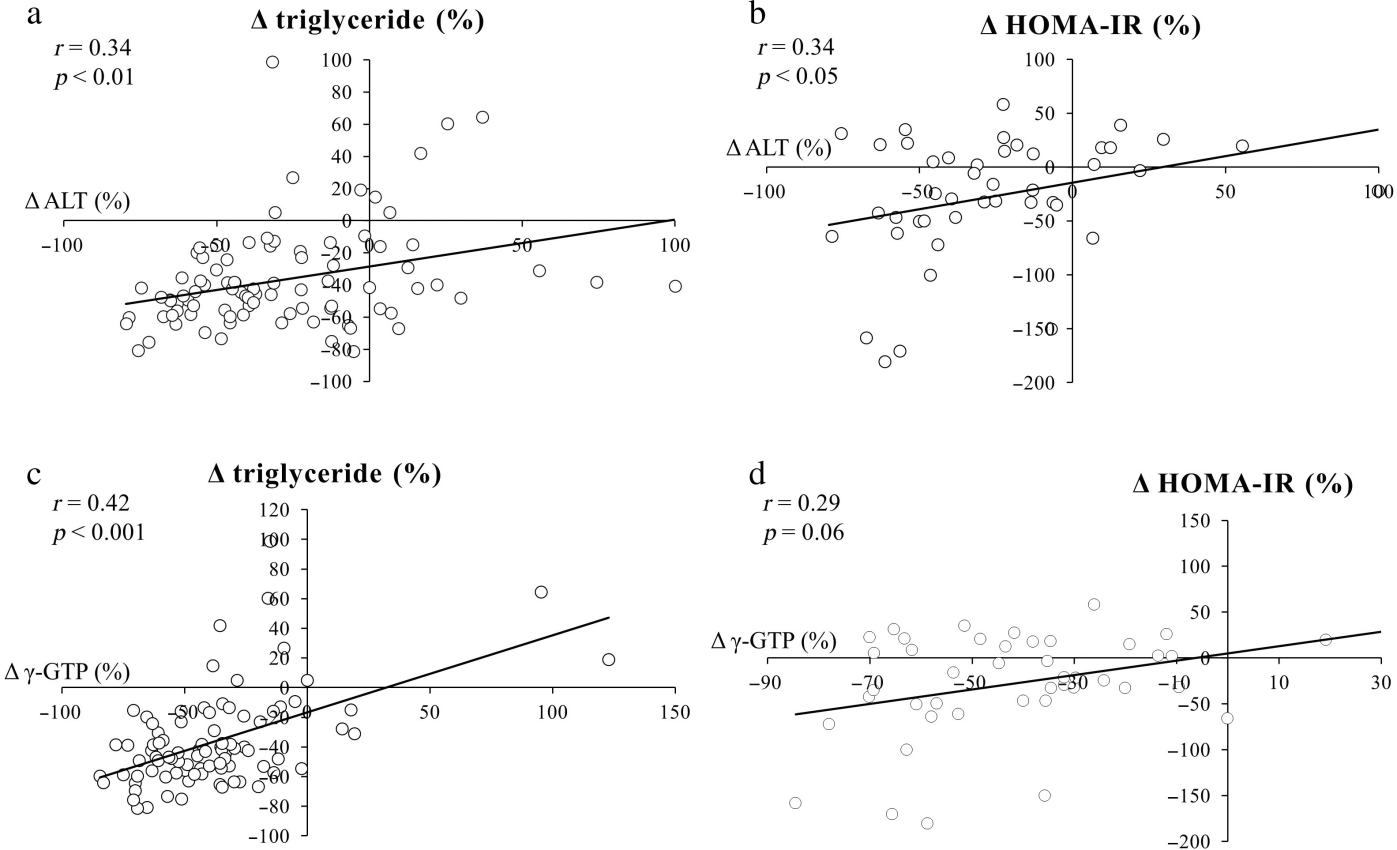
Correlations between changes in alanine aminotransferase (ALT) from baseline to 48 weeks of pemafibrate treatment and in (a) triglyceride and (b) homeostasis model assessment‐insulin resistance (HOMA‐IR). Correlation from baseline to 48 weeks of the treatment between changes in gamma‐glutamyl transpeptidase (γ‐GTP) and in (c) triglyceride and (d) HOMA‐IR.

Moreover, changes from baseline to Week 48 in ALT were weakly correlated with those in triglyceride (*r* = 0.34; *P* < 0.01) and HOMA‐IR (*r* = 0.34; *P* < 0.05). Similarly, changes from baseline to Week 48 in γ‐GTP were correlated with those in triglyceride (*r* = 0.42; *P* < 0.001) (Fig. 3).

In contrast to these laboratory results, CAP values, which reflect lipid accumulation in the liver, did not significantly improve after 48‐week pemafibrate treatment (Table 2). Furthermore, no significant changes in body weight and BMI were observed during the study period (Table 2 and Fig. 1).

### Anti‐fibrotic effect of pemafibrate

**Figure 4:**
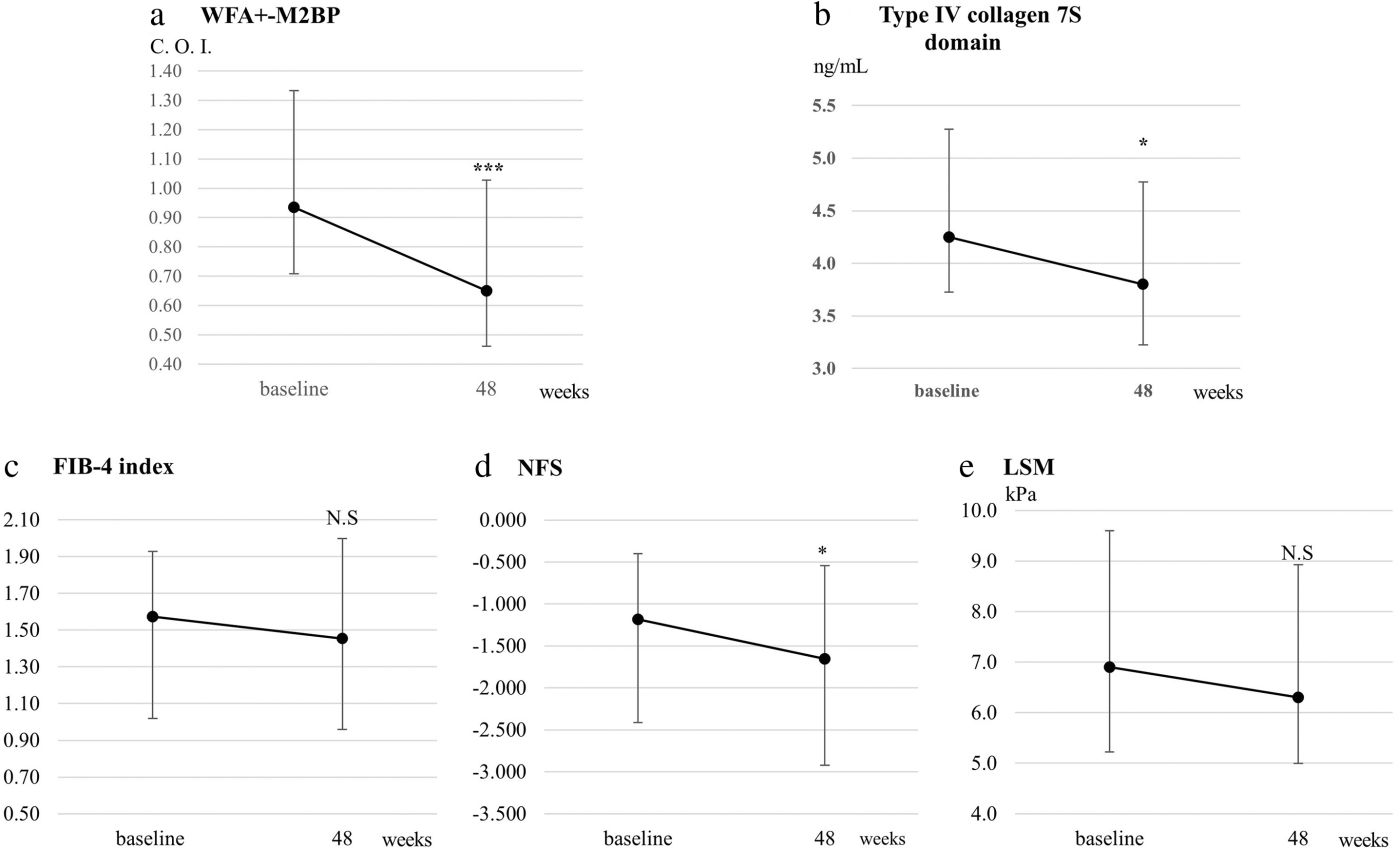
Changes from baseline to 48 weeks of pemafibrate treatment in (a) Wisteria floribunda agglutinin‐positive Mac‐2‐binding protein (WFA^+^‐M2BP), (b) Type IV collagen 7S domain, (c) Fibrosis‐4 (FIB‐4) index, (d) NAFLD (nonalcoholic fatty liver disease) fibrosis score (NFS), and (e) liver stiffness measurement (LSM). Error bars denote interquartile ranges. *P < 0.05 *versus* baseline. ***P < 0.001 *versus* baseline.

The median levels of WFA^+^‐M2BP and type IV collagen 7s significantly decreased from 0.94 C.O.I. and 4.3 ng/mL at baseline to 0.65 C.O.I. and 3.8 ng/mL at Week 48, respectively (*P* < 0.001 and <0.05, respectively; Table 2 and Fig. 4). NFS also significantly decreased from −1.183 at baseline to −1.655 (*P* < 0.01). The FIB‐4 index decreased from 1.57 at baseline to 1.45 at Week 48, although with no statistical significance (*P* = 0.07). LSM, which reflects the degree of liver fibrosis, did not significantly decrease at Week 48 (*P* = 0.13) (Table 2 and Fig. 4).

As for changes in platelet and albumin, both significantly increased from baseline to Week 48 (*P* < 0.001 and <0.05, respectively; Table 2). Specifically, platelets and albumin levels markedly increased in the advanced fibrosis group (165 × 10^3^/μL at baseline to 193 × 10^3^/μL at Week 48; *P* < 0.001) and the low‐albumin group (3.9 g/dL at baseline to 4.2 g/dL at Week 48; *P* < 0.01) (Fig. S2).

### Safety

Of the 110 patients who received pemafibrate, 24 (21.8%) showed adverse events. Twenty‐one patients (19.1%) completed the 48‐week pemafibrate treatment as scheduled, whereas three (2.7%) patients discontinued the treatment prematurely due to nausea (Grade 1), discomfort (Grade 1), and myalgia (Grade 2), respectively (Table 3). Most adverse events were transient, ranging from Grade 1 (mild) to Grade 2 (moderate). Only one case presented with a Grade 3 (severe) adverse event (exacerbated preexisting heart failure); however, this was not drug‐related, and therefore, the 48‐week treatment was completed for this case. No life‐threatening or fatal adverse events were observed during the treatment period.

**Table 3:** Treatment adverse events

Adverse events	Patients who completed the treatment (*n* = 91)	Patients who discontinued the treatment (*n* = 19)
Grade 4–5 adverse event	0 (0)	0 (0)
Grade 3 adverse event	1 (0.9)	0 (0)
Heart failure	1 (0.9)	
Grade 1–2 adverse events	30 (27)	3 (2.7)
Dizziness	3 (2.7)	
Hypertension	3 (2.7)	
Hyperuricemia	3 (2.7)	
Constipation	2 (1.8)	
Cough	2 (1.8)	
Nausea	1 (0.9)	1 (0.9)
Pedal edema	2 (1.8)	
Elevation of serum creatinine	2 (1.8)	
Discomfort		1 (0.9)
Anemia	1 (0.9)	
Pruritus of hands	1 (0.9)	
Fever	1 (0.9)	
Pharyngitis	1 (0.9)	
Numbness of foot	1 (0.9)	
Spinal compression fracture	1 (0.9)	
Asthma	1 (0.9)	
Atrial fibrillation	1 (0.9)	
Hyperkalemia	1 (0.9)	
Elevation of serum creatine kinase	1 (0.9)	
Abdominal pain	1 (0.9)	
Myalgia		1 (0.9)
Eosinophilia	1 (0.9)	

Data are presented as numbers (percentages).

## Discussion

Phase 2 dose‐finding and randomized, controlled Phase 3 trials reported that 12‐ and 24‐week pemafibrate (0.1 mg twice daily) administration decreased liver enzyme levels, ALT and γ‐GTP, in 36 and 73 patients with dyslipidemia (triglyceride ≥200 or 150 mg/dL and HDL‐C < 50 mg/dL), respectively, including those with mild liver injury (AST or ALT <2 times the upper limit of normal).^29^, ^30^ A pilot study of 20 patients with MASLD complicated by hypertriglyceridemia (triglyceride ≥150 mg/dL) suggested that 12‐week pemafibrate administration decreased these liver enzyme levels.^23^ Subsequently, few retrospective studies reported similar pemafibrate effects in patients with MASLD complicated dyslipidemia. One retrospective study reported significant decreases in ALT, ALP, and γ‐GTP with pemafibrate for 3 months in 38 patients.^24^ Another two retrospective studies reported these pemafibrate effects throughout the 48‐week study period in 31 and 138 patients, respectively, with MASLD complicated by hypertriglyceridemia (fasting triglyceride ≥150 mg/dL or non‐fasting triglyceride ≥175 mg/dL).^25^, ^26^ The present prospective study confirmed the effectiveness of 48‐week pemafibrate treatment in 91 patients with MASLD complicated dyslipidemia: pemafibrate improved liver enzyme levels (ALT, γ‐GTP, and ALP) and lipid metabolism, without changes in body weight/BMI between the first 12 and 48 weeks of treatment. Additionally, this study yielded the following notable findings: (i) significant correlations between changes in ALT or γ‐GTP and in triglyceride; and (ii) an ameliorative effect of pemafibrate on insulin resistance without changes in body weight/BMI in patients without diabetes mellitus.

Given that increased ALT and γ‐GTP levels reflect liver injury and inflammation,^38^ the aforementioned studies suggest that pemafibrate improves and suppresses these pathological conditions. The present study revealed that changes in ALT and γ‐GTP were significantly correlated with those in triglyceride, indicating that improvement in lipid metabolism with pemafibrate contributes to improving liver injury and inflammation. However, a double‐blind, placebo‐controlled, randomized trial on patients with MASLD revealed that pemafibrate improved liver inflammation without a reduction in hepatic lipid accumulation on magnetic resonance elastography (MRE).^22^ Similarly, this study failed to show a pemafibrate‐induced decrease in the CAP value, which is employed as an index of lipid accumulation in the liver.^39^ These findings suggest that pemafibrate ameliorates liver inflammation through a mechanism that does not involve a reduction in intrahepatic lipid content. An in vivo study using the STAM MASH mouse model demonstrated that pemafibrate reduced large lipid droplets, ballooning, and NAFLD activity score without decreasing triglyceride accumulation in the liver.^41^ One possible rationale for the lack of intrahepatic triglyceride reduction is that pemafibrate enhances intrahepatic triglyceride reduction and β‐oxidation while promoting the re‐esterification from dihydroxyacetone 3‐phosphate and monoacylglycerol to triglyceride. In this process, free fatty acid in the liver decreased, possibly alleviating lipotoxicity and consequently improving liver inflammation. Alternatively, given that large lipid droplets are involved in the onset of liver inflammation in patients with MASLD,^40^ a reduction in these large lipid droplets may have contributed to the improvement in liver inflammation. Additionally, a decrease in inflammation‐related genes, molecules, and biomarkers noted in the STAM mice suggested direct ameliorating effects of pemafibrate on inflammation. Furthermore, a study using an amylin liver MASH mouse model demonstrated that pemafibrate reduced histological steatosis and ballooning, suggesting that increased lipid turnover may have reduced steatosis and liver inflammation.^41^

A subgroup analysis of a Phase 3 trial involving patients with type 2 diabetes mellitus and hypertriglyceridemia revealed that pemafibrate decreased the HOMA‐IR level and improved insulin resistance through an increase in the serum fibroblast growth factor 21 level.^31^ Moreover, the present study demonstrated that changes in ALT were significantly correlated with those in HOMA‐IR in patients without diabetes mellitus, suggesting that an improvement in insulin resistance, a known trigger for the onset/progression of MASLD,^42^ may contribute to the amelioration of liver injury.

Comparing the characteristics of responders and non‐responders to pemafibrate treatment in this study, baseline liver enzyme levels such as AST, ALT, and γ‐GTP were significantly higher in responders than in non‐responders. This result is generally in line with a previously reported result: In a retrospective analysis of 75 patients with MASLD complicated by dyslipidemia using the FibroScan‐AST (FAST) score as a measure of treatment response, baseline γ‐GTP emerged as an independent predictor of the response to pemafibrate treatment.^43^ These findings suggest that pemafibrate treatment may offer greater efficacy in MASLD patients with higher liver enzyme levels, providing a rationale for selecting pemafibrate in such patients.

Liver stiffness evaluated using MRE and WFA^+^‐M2BP in the pemafibrate‐treated group was significantly lower than in the placebo group in the randomized clinical trial.^22^ An animal experiment using mice showed a pemafibrate‐administration‐related improvement in liver fibrosis, with the suppression of intrahepatic pro‐inflammatory gene and collagen 1α1 mRNA expression.^41^ The present study demonstrated that 48‐week pemafibrate administration significantly improved liver fibrosis biomarkers, such as platelets, NFS, WFA^+^‐M2BP, and type IV collagen 7S, but not LSM and FIB‐4 index. The absence of a significant decrease in FIB‐4 index could possibly be attributed to a decrease in the ALT level was more marked than that in the AST level. As a clinically crucial point, pemafibrate markedly improved respective parameters in patients with a low platelet count or albumin level. To date, there are no approved therapies to improve liver fibrosis; therefore, pemafibrate may be useful in improving or suppressing liver fibrosis in patients with MASLD complicated by dyslipidemia. However, due to the nature of this study being a clinical study, patients without advanced fibrosis were included in the study. Thus, it was unclear whether liver fibrosis was improved by pemafibrate treatment in patients at high risk for liver fibrosis. Therefore, further investigation is needed to determine whether pemafibrate improves liver fibrosis in patients with advanced or significant liver fibrosis.

This study has some limitations. First, histological evaluation via liver biopsy was not conducted to investigate time‐course changes in liver inflammation and fibrosis. Accordingly, changes in lipid droplets and intrahepatic lipid content remain unclear. Second, the glucose clamp test was not performed to assess insulin sensitivity or resistance. Only HOMA‐IR was evaluated in a portion of this study population (i.e. only patients without drug‐treated diabetes), given that it cannot be accurately assessed during the administration of anti‐diabetic agents. Therefore, insulin resistance improvement with pemafibrate may provide an unknown bias. Third, it remains unclear whether pemafibrate could exert similar effects in patients without dyslipidemia or with other lipidemia types. Lastly, the relatively short observation period of 48 weeks suggests that short‐term events may be influenced by the preexisting conditions before pemafibrate administration. A long‐term, large‐scale study is needed to determine the utility of pemafibrate in patients with MASLD, specifically, a reduction in intrahepatic lipid accumulation.

In conclusion, 48‐week pemafibrate treatment improved liver inflammation, as well as lipid metabolism, where changes in liver enzymes significantly correlated with those in triglyceride and HOMA‐IR, and might improve liver fibrosis in patients with MASLD complicated by dyslipidemia. Further validation is required regarding the synergistic effects of pemafibrate in combination with antidiabetic agents with potential effects on MASLD, such as SGLT2‐Is and GLP‐1 RAs, which were commonly used for type 2 diabetes mellitus in this population.

## Ethics statement

This study was performed in accordance with the ethical guidelines of the 2013 Declaration of Helsinki and was approved by the Ethics Committee of Nippon Medical School Hospital (approval number: B‐2020‐319).

## Patient consent statement

All patients provided written informed consent prior to study inclusion.

## Supporting information


**Figure S1.** Correlations between changes in body mass index (BMI) from baseline to 48 weeks of pemafibrate treatment and in (a) alanine aminotransferase (ALT), (b) gamma‐glutamyl transpeptidase (γ‐GTP), and (c) homeostasis model assessment‐insulin resistance (HOMA‐IR).

**Figure S2.** Changes in platelets from baseline to 48 weeks of pemafibrate treatment in (a) patients with platelet <192 × 10^3^/μL at baseline and (b) patients with platelets ≥192 × 10^3^/μL. Changes in albumin from baseline to 48 weeks of treatment in (a) patients with albumin ≤4 g/dL at baseline and (b) patients with albumin >4 g/dL. Error bars denote interquartile ranges. **P < 0.01 *versus* baseline. ***P < 0.001 *versus* baseline.

**Table S1.** Comparison of responders and non‐responders to pemafibrate treatment at baseline.

## Data Availability

The data that support the findings of this study are available from the corresponding author, Masanori Atsukawa, upon reasonable request.
