# Supplementary figures and images for: Impact of pemafibrate in patients with metabolic dysfunction‐associated steatotic liver disease complicated by dyslipidemia: A single‐arm prospective study
Source: JGH Open. 2024 Apr 2;8(4):e13057. doi: 10.1002/jgh3.13057 (PMC10986296; doi:10.1002/jgh3.13057)

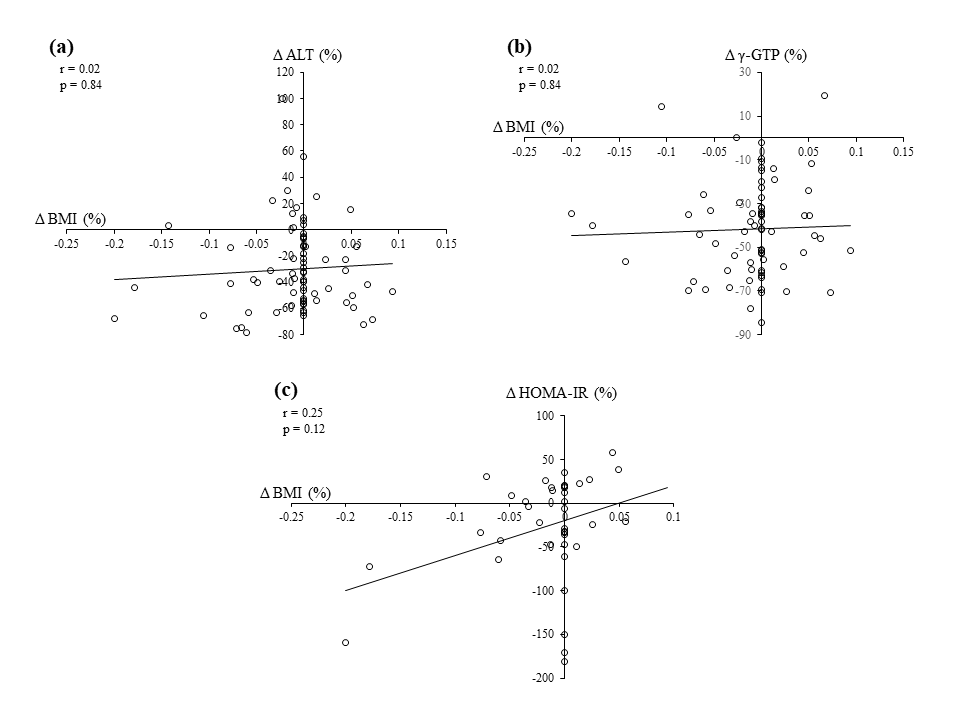

Supplement: Supplementary file 1 — Figure S1. Correlations between changes in body mass index (BMI) from baseline to 48 weeks of pemafibrate treatment and in (a) alanine aminotransferase (ALT), (b) gamma‐glutamyl transpeptidase (γ‐GTP), and (c) homeostasis model assessment‐insulin resistance (HOMA‐IR). [file JGH3-8-e13057-s002.tif]

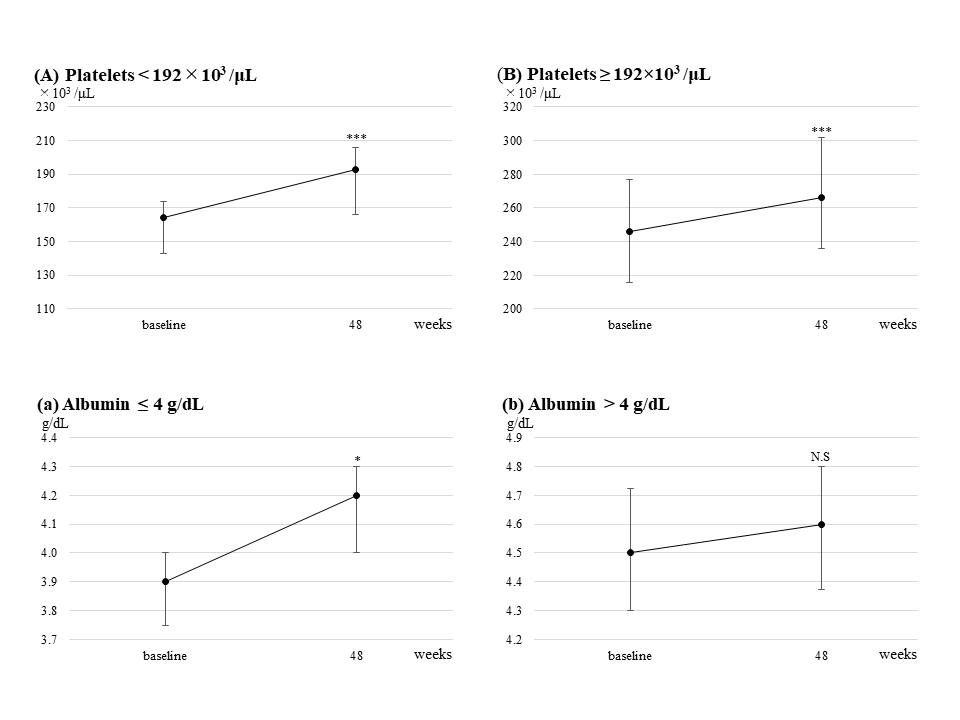

Supplement: Supplementary file 2 — Figure S2. Changes in platelets from baseline to 48 weeks of pemafibrate treatment in (a) patients with platelet <192 × 103/μL at baseline and (b) patients with platelets ≥192 × 103/μL. Changes in albumin from baseline to 48 weeks of treatment in (a) patients with albumin ≤4 g/dL at baseline and (b) patients with albumin >4 g/dL. Error bars denote interquartile ranges. **P < 0.01 versus baseline. ***P < 0.001 versus baseline. [file JGH3-8-e13057-s001.tif]
