# Supplementary material for: Impact of pemafibrate in patients with metabolic dysfunction‐associated steatotic liver disease complicated by dyslipidemia: A single‐arm prospective study
Source: JGH Open. 2024 Apr 2;8(4):e13057. doi: 10.1002/jgh3.13057 (PMC10986296; doi:10.1002/jgh3.13057)
Supplement: Supplementary file 3 — Table S1. Comparison of responders and non‐responders to pemafibrate treatment at baseline. [file JGH3-8-e13057-s003.docx]

Supplementary Table 1. Comparison of responders and non-responders to pemafibrate treatment at baseline

| Factors | Responders | Non-responders | p value |
| --- | --- | --- | --- |
| Age (year) | 60 (51–72) | 63 (55–70) | 0.61 |
| Gender (Male/Female) | (31/23) | (23/14) | 0.67 |
| Body weight (kg) | 73.0 (62.1–80.0) | 68.0 (60.5–84.8) | 0.80 |
| BMI (kg/m^2^) | 27.3 (24.5–30.2) | 26.4 (23.5–30.0) | 0.48 |
| Platelets (×10^3^/mm^3^) | 216 (167–259) | 215 (191–255) | 0.55 |
| AST (U/L) | 40 (33–59) | 34 (24–40) | < 0.01 |
| ALT (U/L) | 62 (36–90) | 43 (29–62) | < 0.01 |
| γ-GTP (U/L) | 71 (47–168) | 42 (26–71) | < 0.01 |
| ALP (U/L) | 130 (73–235) | 106 (68–220) | 0.75 |
| Serum albumin (g/dL) | 4.5 (4.2–4.7) | 4.4 (4.3–4.8) | 0.47 |
| Triglyceride (mg/dL) | 207 (162–275) | 191 (150–249) | 0.37 |
| Total cholesterol (mg/dL) | 228 (193–252) | 217 (192–250) | 0.19 |
| LDL cholesterol (mg/dL) | 142 (113–169) | 128 (108–167) | 0.44 |
| HDL cholesterol (mg/dL) | 45 (40–57) | 49 (40–54) | 0.84 |
| HbA1c (%) | 6.3 (5.8–7.3) | 6.1 (5.7–6.5) | 0.12 |
| Fasting Plasma glucose (mg/dL) | 128 (104–155) | 109 (99–127) | 0.06 |
| IRI (μU/mL) | 13.6 (9.6–21.5) | 13.3(10.0–18.1) | 0.56 |
| HOMA-IR | 3.96 (2.78–5.06) | 3.59 (2.33–5.46) | 0.54 |
| Type Ⅳ collagen 7S domain (ng/mL) | 4.5 (3.9–6.2) | 4.2 (3.7–5.1) | 0.21 |
| WFA^+^-M2BP (C.O.I.) | 0.93 (0.60–1.38) | 0.93 (0.76–1.21) | 0.98 |
| FIB-4 index | 1.59 (1.04–2.01) | 1.53 (1.01–1.88) | 0.50 |
| NFS | -1.159  (-2.370–-0.4110) | -1.438  (-2.466–-0.512) | 0.46 |
| LSM (kPa) | 6.7 (4.6–10.7) | 6.2 (5.0–9.0) | 0.77 |
| CAP (dB/m) | 319 (273–351) | 297 (270–344) | 0.47 |

Data are presented as numbers or medians (interquartile ranges).

BMI, body mass index; AST, aspartate aminotransferase; ALT, alanine aminotransferase; γ-GTP, gamma glutamyl transpeptidase; ALP, alkaline phosphatase; LDL, low-density lipoprotein; HDL, high-density lipoprotein; HbA1c, hemoglobin A1c; IRI, immunoreactive insulin; HOMA-IR, homeostasis model assessment-insulin resistance; WFA^+^-M2BP, Wisteria floribunda agglutinin positive Mac-2-binding protein; FIB-4 index, fibrosis-4 index; NFS, NAFLD (nonalcoholic fatty liver disease) fibrosis score; LSM, liver stiffness measurement; CAP, controlled attenuation parameter.
